# Controlled synthesis of series Ni_x_Co_3-x_O_4_ products: Morphological evolution towards quasi-single-crystal structure for high-performance and stable lithium-ion batteries

**DOI:** 10.1038/srep11584

**Published:** 2015-06-24

**Authors:** Yu Zhou, Yong Liu, Wenxia Zhao, Hai Wang, Baojun Li, Xiang Zhou, Hui Shen

**Affiliations:** 1 School of Physics and Engineering, State Key Laboratory of Optoelectronic Materials and Technologies, Sun Yat-sen University, Guangzhou 510275, China; 2 Instrumental Analysis & Research Center, Sun Yat-sen University, Guangzhou 510275, China; 3 Key Laboratory of New Processing Technology for Nonferrous Metal and Materials, Ministry of Education, Guilin University of Technology, Guilin, 541004, China

## Abstract

Transition metal oxides are very promising alternative anode materials for high-performance lithium-ion batteries (LIBs). However, their conversion reactions and concomitant volume expansion cause the pulverization, leading to poor cycling stability, which limit their applications. Here, we present the quasi-single-crystal Ni_x_Co_3-x_O_4_ hexagonal microtube (QNHM) composed of continuously twinned single crystal submicron-cubes as anode materials for LIBs with high energy density and long cycle life. At the current density of 0.8 A g^−1^, it can deliver a high discharge capacities of 1470 mAh g^−1^ over 100 cycles (105% of the 2nd cycle) and 590 mAh g^−1^ even after 1000 cycles. To better understand what underlying factors lead our QNHMs to achieve excellent electrochemical performance, a series of Ni_x_Co_3-x_O_4_ products with systematic shape evolution from spherical to polyhedral, and cubic particles as well as circular microtubes consisted of spheres and square microtubes composed of polyhedra have been synthesized. The excellent electrochemical performance of QNHMs is attributed to the unique stable quasi-single-crystal structure, which can both provide efficient electrical transport pathway and suppress the electrode pulverization. It is important to note that such quasi-single-crystal structure would be helpful to explore other high-energy lithium storage materials based on alloying or conversion reactions.

Application targets of lithium-ion batteries (LIBs) are moving from portable electronic devices to their proposed use in high-power systems such as electric drive vehicles or renewable energy storage. However, graphite, the current anode material in commercial LIBs, has relatively low theoretical specific capacity (372 mAh g^−1^)^1^. Therefore, considerable efforts are devoted to explore high-energy lithium storage materials based on alloying or conversion reactions such as Si^2^,^3^, Sn^4^, and transition metal oxides^5^,^6^. Among all the transition metal oxide candidates, binary Co_3_O_4_^7^,^8^,^9^,^10^,^11^,^12^,^13^,^14^ and ternary Ni_x_Co_3-x_O_4_ (0 < x ≤ 1)^15^,^16^,^17^ have become the most explored anode materials for recent years since they can in principle deliver high theoretical capacity up to 890 mAh g^−1^. Most of previous studies discussed the potential advantages of their exposed crystal planes^7^, mesoporous structure with high surface area^8^,^14^,^15^, and one dimensional nanostructures^9^,^10^,^11^,^12^,^13^,^16^,^17^ on LIBs. But unlike classical intercalation electrode materials (*i.e.*, graphite and TiO_2_)^1^, the major challenges for application of transition metal oxides in LIBs are that their special conversion reaction and concomitant volume expansion/contraction cause pulverization of electrode materials, which lead to loss of electrical contacts between adjacent particles and poor cycling stability^18^,^19^,^20^,^21^,^22^,^23^,^24^. One possible way to mitigate these problems is by depositing nanostructured materials onto the suitable carbonaceous supports such as graphene to accommodate their large volume change and improve electronic conductivity^18^,^19^,^20^,^21^. However, to date, few studies have given the evidence that the initial structure of Ni_x_Co_3-x_O_4_ materials could be maintained after severe cycles of conversion reactions even by adopting the strategies above. For instance, Chen *et al*. have proposed a NiCo_2_O_4_ nanoplate-graphene nanosheet structure design, yet small particles induced by pulverization were observed on the graphene after just 40 cycles^21^. In additional, the obtained electrochemical performance is not so good as expected since these approaches do not improve the lattice electronic conductivity or lithium ion diffusion within the active materials. The point contact resistance between nanoparticles and carbon materials is still very large due to their nonintimate contacts. Moreover, relative complex procedures as well as addition of expensive graphene additives limit their applications in commercial products, and these low-capacity carbon additives, which usually occupies as much as 30 vol% of the resulting composite, can also suppress the overall energy density^24^,^25^. Great efforts have been devoted to growing active materials on the conductive current collector substrate with the aim of being free of the binders and carbon conductors as well as buffering the volume changes^26^,^27^. However, this type of electrode still demonstrated poor capacity retention and short cycle life. One possibility is that the active materials might peel off from the substrate due to the lattice mismatch between them and special convention reaction, and thus the electron transport pathway would be seriously interrupted without the help of carbon conductors. Therefore, we should consider taking an unconventional approach to construct much more stable electrode structure so that it can provide an efficient electrical transport pathway and suppress the electrode pulverization during severe cycles.

Here, we present the quasi-single-crystal Ni_x_Co_3-x_O_4_ hexagonal microtubes (QNHMs) composed of continuously twinned single crystal submicron-cubes with the aim of suppressing the electrode pulverization and facilitating electronic transport in LIBs. This unique stable structure provides high reversible capacity and excellent cycling stability. At current density of 0.8 A g^−1^, such QNHM electrode can deliver high discharge capacities of 1470 mAh g^−1^ over 100 cycles (105% of the 2nd cycle) and 590 mAh g^−1^ even after 1000 cycles. In our study, not only the problem of pulverization of Ni_x_Co_3-x_O_4_ electrode could be solved by using this stable quasi-single-crystal structure and without the aid of a carbonaceous matrix for the first time, but also the obtained comprehensive performance is the best among Ni_x_Co_3-x_O_4_ materials to date.

## Results

### Morphological and Structural Characterization

**Figure 1:**
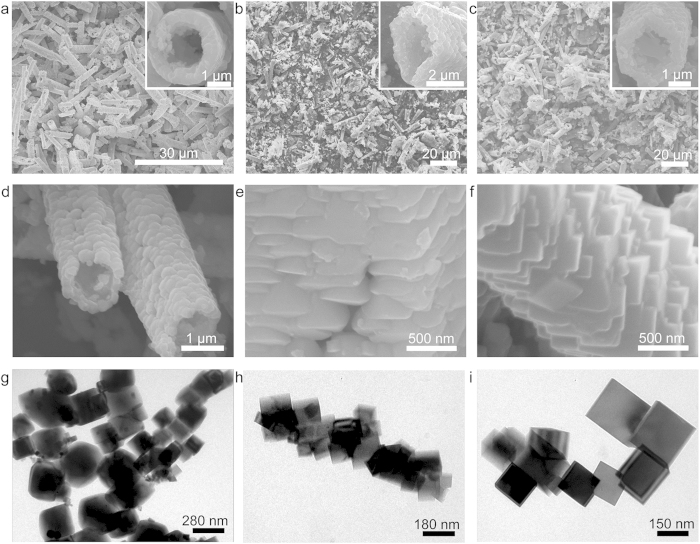
The temperature-dependent morphological evolution of as-synthesized Ni_x_Co_3-x_O_4_ products. Low-magnification SEM images of (**a**) circular microtubes, (**b**) square microtubes, and (**c**) QNHMs prepared by hydrothermal treatment of 0.8 g Ni(NO_3_)_2_∙6H_2_O and 3.2 g Co(NO_3_)_2_∙6H_2_O in 60 mL of ammonia solution (8.3% w/w) for 24 h at 180 °C, 200 °C, and 230 °C, respectively. The insets in (**a**), (**b**) and (**c**) show the tips of three types of microtubes. SEM images of the walls of (**d**) circular microtubes, (**e**) square microtubes, and (**f**) QNHMs. TEM image of the microtube wall structures which derive from (**g**) circular microtubes, (**h**) square microtubes, and (**i**) QNHMs by exfoliating them in intense ultrasonication treatments for 3 h.

**Figure 2:**
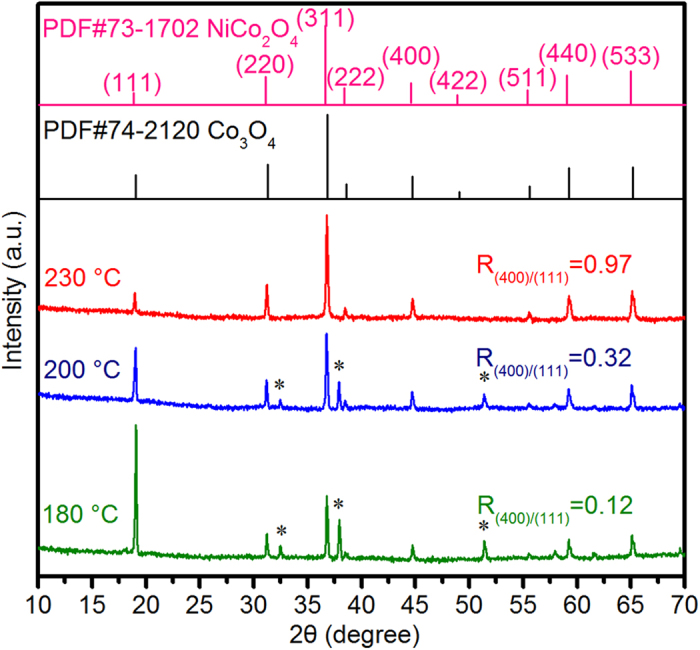
Temperature dependent evolution of XRD measurements. Circular microtubes, square microtubes, and QNHMs were synthesized at hydrothermal temperature of 180 °C, 200 °C, and 230 °C, respectively. Notes: Some peaks marked with asterisk (*) represent β-Co(OH)_2_ phase.

Figure 1 shows the temperature-dependent morphological evolution of as-synthesized Ni_x_Co_3-x_O_4_ samples, from which one can clearly see that the obtained products undergo drastic morphological changes on both micron- and submicron-scale with the increase of temperature. By progressively increasing hydrothermal temperature from 180 °C to 200 °C, and to 230 °C, the morphologies of the products change drastically from circular microtubes to square microtubes, and to final hexagonal microtubes (Fig. 1a–c). The average length/diameter for circular microtubes, square microtubes, and hexagonal microtubes is about 15.9/2.2 μm, 15.1/1.8 μm, and 20.5/2.2 μm, respectively. To further investigate the assemblies of primary building blocks, incomplete microtube walls were obtained by exfoliating them from intense ultrasonication for 3 h, as shown in transmission electron micrscopy (TEM) images (Fig. 1g–i). Combining with the side-view observations of tube walls in enlarged scanning electron microscopy (SEM) images (Fig. 1d–f), the unexpected and unique hierarchical assemblies of building blocks for above microtubes come into our sight. The circular and square microtubes consisted of aggregated irregular submicron-sized spheres or polyhedra, respectively, whereas the quasi-single-crystal Ni_x_Co_3-x_O_4_ hexagonal microtubes (QNHMs) were composed of continuously twinned submicron-cubes. More interestingly, the above morphological evolution of building blocks in these microtubes can be further confirmed by X-ray diffraction (XRD) measurements. Figure 2 shows the XRD patterns of as-synthesized Ni_x_Co_3-x_O_4_ circular microtubes, square microtubes, and QNHMs produced at hydrothermal temperature of 180 °C, 200 °C, and 230 °C, respectively. The crystal phase of the circular and square microtubes is predominantly Ni_x_Co_3-x_O_4_ although small peaks marked with asterisk at 32.5°, 37.9° and 51.4° indicate the formation of trace amounts of brucite-like β-Co(OH)_2_ (JCPDS card no. 30–0443)^28^,^29^. While increasing the hydrothermal temperature to 230 °C, the β-Co(OH)_2_ phase disappeared. All the diffraction peak positions of QNHMs agree well with standard patterns of Co_3_O_4_ (JCPDS no. 74–2120) and NiCo_2_O_4_ (JCPDS no. 73–1702), suggesting the partially substitution of Ni for Co still adopts the cubic spinel structure with almost same lattice constants and thus does not modify the general aspect of XRD patterns. Interestingly, the ratio of the intensity of the (400) peak to that of (111) peak increased from 0.12 for circular microtubes to 0.32 for square microtubes, and to 0.97 for QNHMs, indicating the building blocks for microtubes were evolved from particles with mostly exposed (111) plane into cubes with exposed (400) plane with the increase of temperature from 180 °C to 230 °C. The trend of ratio of intensity relating to the exposed crystal planes here is similar to that of previous reported texture in Cu_2_O and Pt samples^30^,^31^. The X-ray photoelectron spectroscopy (XPS) patterns and corresponding Gaussian fitting further confirm the existence of the Ni, Co, and O elements in QNHMs^15^,^32^,^33^, and an atomic ratio of Ni to Co of 1 : 3.6 was detected, which is very close to our original precursors with Ni to Co ratio of 1:4 (Supplementary Fig. S1).

**Figure 3:**
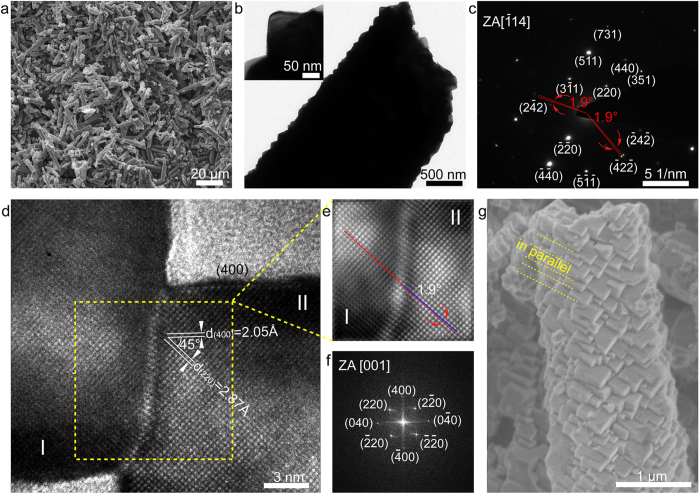
Structural characterizations of the annealed QNHMs. (**a**) SEM images showing a large area view of the annealed QNHMs. (**b**) TEM image of the annealed QNHMs, and the inset demonstrating enlarged QNHM constructed by submicron cubes. (**c**) Corresponding SAED pattern. (**d**) HRTEM image of twinned submicron cubes. (**e**) A Fourier-filtered image and (**f**) FFT image from the region outlined by dashed yellow line in (**d**). (**g**) Side view SEM image of tube walls of annealed QNHMs.

Figure 3 and Supplementary Fig. S2 show the morphological and structural characterizations of QNHMs obtained after annealing at 550 °C for 3 h. It can be clearly seen from the SEM image (Fig. 3a), TEM image (Fig. 3b), and XRD measurement (Supplementary Fig. S2), the overall morphology and crystal structure of QNHMs can be well preserved after the annealing treatment. In addition, the ratio of the intensity of the (400) peak to that of the (111) peak is 1.07 for calcined QNHMs, which is close to that of as-synthesized QNHMs (0.97 calculated in Fig. 2), indicating the texture of the QNHMs are unchanged after the heat treatment. Liu, *et al*. have demonstrated that the strongly textured nanolaminated structure with low-angle boundaries (1–8°) among the lamellae in Nickel was ultra-hard and ultra-stable^34^. Here, it can be seen from the selected area electron diffraction (SAED) pattern (Fig. 3c), most of the diffraction spots are very sharp that is close to patterns of the single crystal diffraction, and the measured angles of 1.9° for splitting (422) spots are very small, indicating that submicron cubes are twinned almost in parallel to build a very stable quasi-single-crystal structure. The High-resolution TEM (HRTEM) observations were performed in order to identify the characteristics of boundaries between adjacent submicron cubes (Fig. 3d), we can see that submicron cubes were twinned together as primary building blocks to construct the quasi-single-crystal hexagonal microtubes. Moreover, the average distance between the adjacent lattice planes is 2.87 Å and 2.05 Å, corresponding to (220) and (400) lattice planes of Ni_x_Co_3-x_O_4_ phase, respectively, which further prove that twinned cubes expose {100} planes. From the Fourier-filtered image (Fig. 3e) and fast Fourier transformation (Fig. 3f) taken from square area in Fig. 3d, we can see that twinned cubes along their boundary demonstrated an inclination by 1.9° between (220) planes in I and II, which is accordance with the SAED analysis. In addition, the side-view of a typical annealed QNHM in SEM image (Fig. 3g) also reveals that the submicron-cubes are almost in parallel and continuously twinned into such unique quasi-single-crystal structures, which would be beneficial to electronic transport and structural stability.

### The Time-Dependent Morphology Evolution and Growth Mechanism

**Figure 4:**
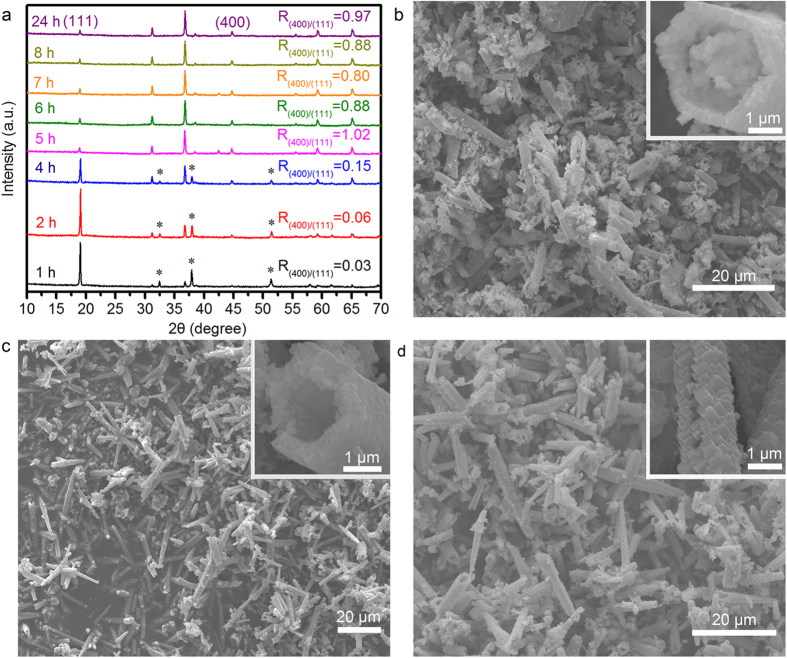
Time dependent morphological and crystal structure evolution of QNHMs. The samples were fabricated by hydrothermal treatment of 0.8 g Ni(NO_3_)_2_∙6H_2_O and 3.2 g Co(NO_3_)_2_∙6H_2_O in 60 mL of ammonia solution (8.3%w/w) at 230 °C for 1–24 h. (**a**) XRD measurements at various stages, and here some peaks marked with asterisk (*) represent β-Co(OH)_2_ phase. (**b**), (**c**) and (**d**) Typical SEM images of QNHMs at the reaction time of 1 h, 2 h, and 5 h, respectively.

To gain more insight into the growth mechanism of QNHMs, the time-dependent experiments were also performed at the hydrothermal temperature of 230 °C with the reaction time changing from 1 h to 24 h, as shown in Fig. 4. While the reaction time was less than 5h, the crystal phase is predominantly Ni_x_Co_3-x_O_4_, and trace amounts of brucite-like β-Co(OH)_2_ whose peaks at 32.5° and 37.9°, and 51.4° were also detected. After 5 h of reaction, the β-Co(OH)_2_ impurity disappeared and only a pure Ni_x_Co_3-x_O_4_, phase was produced, which illustrate that β-Co(OH)_2_ is the intermediate product, and it would completely decomposed to water and Ni_x_Co_3-x_O_4_ crystal with the increase of the reaction time. Surprisingly, similar to temperature-dependent evolution of Ni_x_Co_3-x_O_4_ products (see Fig. 2), time-dependent XRD measurements (Fig. 4a) show that the ratio of the intensity of (400) peak to that of (111) peak increased from 0.03 at 1 h to 0.06 at 2 h, to 0.15 at 4 h, and to 1.02 at 5 h with the increase of reaction time, indicating the building blocks for hexagonal microtubes were also evolved from particles with mostly exposed {111} plane to cubes with exposed {100} plane. After 5 h of reaction, the ratio of the intensity of (400) peak to that of the (111) peak is in the range of 0.8–1.0, revealing the texture of QNHMs were almost unchanged. Time dependent SEM images of products also confirm that the primary building blocks for hexagonal tubes are transformed from the particles exposed {111} plane to cubes exposed {100} plane with the increase of reaction time (Fig. 4b–d). At the early reaction stage of 1 h, large scale and well separated hexagonal microtubes containing nanoparticles inside began to form (Fig. 4b and the inset). With an increase of reaction time up to 2 h, the nanoparticles inside the microtubes disappeared and the tube wall obviously became thick (Fig. 4c and the inset). When the growth time was further extended to 5 h, the building blocks for microtubes were developed into well crystallized submicron-cubes (Fig. 4d and the inset). Based on the above experiments, we propose a possible growth process involving multi-step formation of QNHMs. Initially, Ni and Co precursor was dissolved in ammonia solution, resulting in the formation of a lot of nucleus. Subsequently, many nanocrystals aggregated into hexagonal tubular structures with the help of fast out-diffusion of NH_3_ bubbles *via* the decomposition of ammonia solution under the hydrothermal condition. With the increase of reaction time, nanoparticles inside the hexagonal microtube disappeared, and then the tube wall became thick following the Ostwald ripening process^35^. Finally, the building blocks of hexagonal microtubes evolved from the particles with mostly exposed {111} plane to cubes with exposed {100} plane via the dissolution and recrystallization process.

### Electrochemical Performance

**Figure 5:**
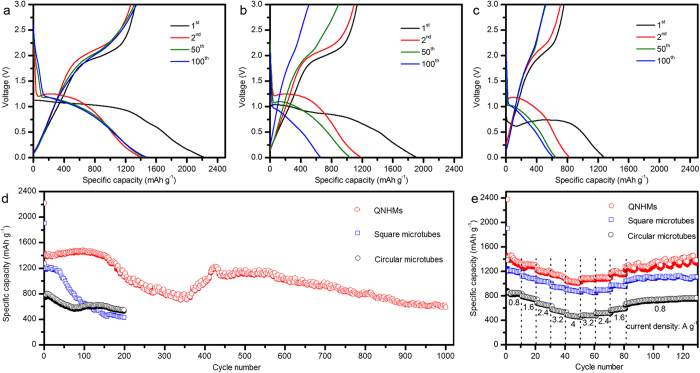
Electrochemical performances of LIBs based on QNHMs, square microtubes, and circular microtubes. Galvanostatic charge-discharge curves of (**a**) QNHMs, (**b**) square microtubes, and (**c**) circular microtubes cycled at the 1st, 2nd, 50th and 100th between 3 and 0.01 V (*vs* Li/Li^+^) at a current density of 0.8 A g^−1^. (**d**) The cycling performance of QNHMs, square microtubes, and circular microtubes at a current density of 0.8 A g^−1^. (**e**) Rate capability of QNHMs, square microtubes, and circular microtubes.

Figure 5a–c show the selected discharge and charge profiles of the electrodes at a current density of 0.8 A g^−1^ over the potential range of 0.01–3.0 V. The first discharge capacities of QNHM electrode was 2218 mAh g^−1^, which was much larger than those of square microtube electrode (1903 mAh g^−1^) and circular microtube electrode (1276 mAh g^−1^). After the second cycle, QNHM electrode also presented much better electrochemical lithium storage performance and cycling stability than others. For instance, the discharge capacities of QNHM electrode were 1399 mAh g^−1^, 1442 mAh g^−1^, and 1470 mAh g^−1^ in the 2^nd^, 50^th^, 100^th^ cycle, respectively. In contrast, the discharge capacities decreased to 1187 mAh g^−1^ for square microtube and 794 mAh g^−1^ for circular microtube after 2^nd^ cycle, and then quickly dropped to 652 mAh g^−1^ for square microtube and 615 mAh g^−1^ for circular microtube in 100^th^ cycle. The cycle performance of electrodes was further investigated at a current density of 0.8 A g^−1^ and obviously QNHM electrode demonstrated the best cycle stability (Fig. 5d). Surprisingly, even after 1000 cycles, the QNHM electrode still retained a discharge capacity of 590 mAh g^−1^. In addition, the QNHM electrode showed enhanced rate capacity in comparison with square microtube and circular microtube electrodes (Fig. 5e). The QNHM electrode could deliver high discharge capacity of 1400, 1300, 1200, 1150, 1100 mAh g^−1^ when the current density changed stepwise from 0.8 to 1.6, 2.4, 3.2, and 4.0 A g^−1^, respectively. More importantly, after this high-rate charge-discharge process, a stable capacity of 1350 mA g^−1^ could be supplied again when the current rate was reduced back to 0.8 A g^−1^, indicating great potential as the high rate electrode materials. It should be noted that the capacities of QNHMs reported here are higher than the theoretical value (890 mAh g^−1^), which was generally observed in the transitional metal oxide electrode studies^6^,^14^,^36^,^37^,^38^. As discussed in previous literatures, the extra capacity is attributed to “pseudo-capacitive behavior”, which originates from the reversible formation/decomposition of polymergic/gel-like film on the surface of active materials during the discharge/charge processes^15^,^39^,^40^,^41^. Meanwhile, the large excess capacities of QNHMs were reversible and well preserved during long-term discharge/charge cycles. According to the observation and detail discussion in following paragraph, QNHMs can retain good structural integrity upon cycling that is favorable for the ease and access of ions and electrons in the Li_2_O/Co matrix. The discharge capacity retention and cycling stability for QNHMs reported here are higher than that of previously reported Co_3_O_4_ or Ni_x_O_3-x_O_4_ electrodes with exposed specific crystal planes^6^, high surface area^8^,^14^,^15^, one dimensional structures^9^,^10^,^11^,^12^,^13^,^16^,^17^, and even that treated with graphene supports^18^,^19^,^20^,^21^,^42^,^43^ while tested under similar conditions. To the best of our knowledge, this is the first time the successful synthesis of quasi-single-crystal Ni_x_Co_3-x_O_4_ structure and maybe one of the best performances among Ni_x_Co_3-x_O_4_ based LIBs.

## Discussion

**Figure 6:**
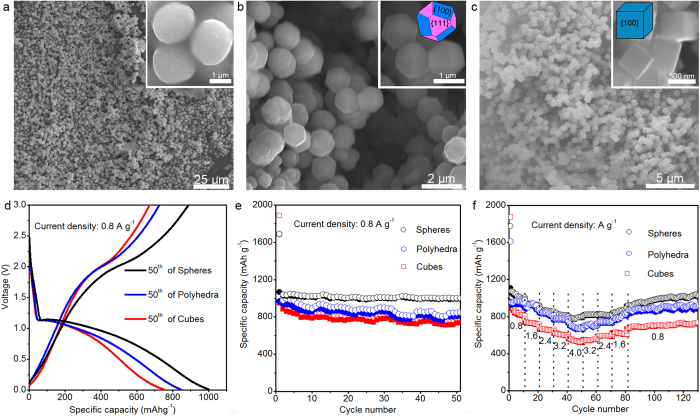
The morphologies of monodisperse Ni_x_Co_3-x_O_4_ particles and their Electrochemical performances. Low-magnification SEM images of (**a**) spheres, (**b**) polyhedra, and (**c**) cubes prepared by hydrothermal treatment of 0.4 g Ni(NO_3_)_2_∙6H_2_O, 1.6 g Co(NO_3_)_2_∙6H_2_O and 0.1 g PVP in 60 mL of 25% (w/w) ammonia solution at 230 °C for 3 h, 12 h and 24 h, respectively. The insets in (**a**), (**b**) and (**c**) showing enlarged monodispersed spheres, polyhedra, and cubes, respectively. (**d**) Galvanostatic discharge-charge voltage profiles of monodispersed spheres, polyhedra, and cubes in the 50th cycle at a current density of 0.8 A g^−1^. (**e**) The cycling performance of monodispersed spheres, polyhedra, and cubes at a current density of 0.8 A g^−1^. (**f**) Rate performance of monodispersed spheres, polyhedra, and cubes at various current densities.

Xiao and co-workers have reported that Co_3_O_4_ particles with {111} planes exhibited better electrochemical performance than that with {100} planes^7^. Many previous researchers also think that mesoporous and nanostructure features with a large surface area would offer sufficient electrochemical reaction sites in the electrode^8^,^14^,^15^. As we discussed previously, the building blocks for circular microtubes, square microtubes, and QNHMs are evolved from particles with mostly exposed {111} plane to cubes with exposed {100} plane. In addition, the measured specific surface area is 19.2 m^2^ g^−1^ for circular microtubes, and then decreased to 3.0 m^2^ g^−1^ for square microtubes and 2.1 m^2^ g^−1^ for QNHMs (Supplementary Fig. S3). Contrary to our expectations, QNHMs achieved excellent electrochemical performance and the best cycle stability even though they exhibited very small specific surface area and exposed {100} crystal plane that are unfavourable to the electrochemical performance. Then we should consider what the underlying factor that reverses the previous results is? Firstly, if we could also synthesize monodisperse Ni_x_Co_3-x_O_4_ particles with exposed planes similar to the building blocks of the above three types of microtubes, it would help us to better understand whether quasi-single-crystal structure or exposed crystal planes indeed affects the electrochemical performance of our QNHMs. Interestingly, a series of monodisperse Ni_x_Co_3-x_O_4_ particles with systematic shape evolution from spheres to polyhedra and cubes were synthesized by justly decreasing concentration of Ni and Co precursor, increasing ammonia concentration, adding PVP surfactant, and changing reaction time as compared with the reaction condition of QNHMs. Figure 6a presents the typical SEM images of Ni_x_Co_3-x_O_4_ spherical particles with a diameter of about 1.8 μm. Moving to Fig. 6b and Supplementary Fig. S4a and b, we can see that particles demonstrate polyhedral morphology with a size of about 1.0–1.2 μm, and they are consisted by eight {111} planes and six {100} planes. As shown in Fig. 6c, Supplementary Fig. S4c and d, the size of Ni_x_Co_3-x_O_4_ cubes is about 490 nm and cubes expose six {100} planes. Therefore, prolonging reaction time may be beneficial for improving the growth rate along <100> to that of <111> of Ni_x_Co_3-x_O_4_ particles, and promote the formation of Ni_x_Co_3-x_O_4_ cubes with {100} planes^44^. In addition, the measured specific surface area for spheres, polyhedral, and cubes is 1.57 m^2^ g^−1^, 1.22 m^2^ g^−1^, and 1.97 m^2^ g^−1^, respectively. The specific surface area is so close that we can ignore their effects on the electrochemical performance. As shown in Fig. 6d-f, the Ni_x_Co_3-x_O_4_ cubes with exposed {100} crystal plane exhibited the worst electrochemical performance among these Ni_x_Co_3-x_O_4_ particles, which is consistent with the previous studies^7^. Our QNHMs also expose {100} crystal plane, but they give rise to the highest lithium storage performance among these Ni_x_Co_3-x_O_4_ materials, which can be attributed to their unique quasi-single-crystal structure ensuring fast electron transfer and high Li^+^ ion diffusion rates.

**Figure 7:**
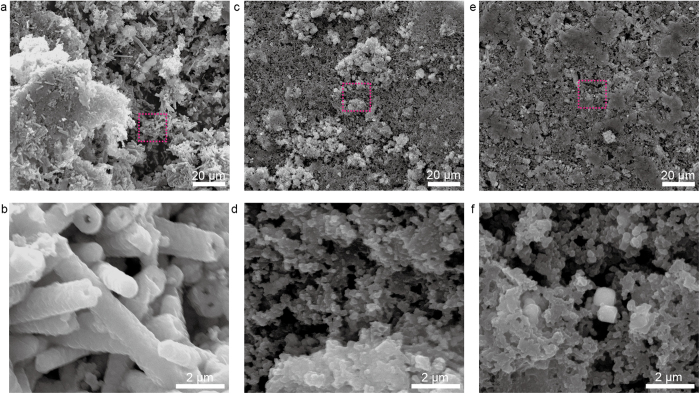
SEM characterization of (a and b) QNHM, (c and d) square microtube, and (e and f) circular microtube electrodes after completions of 100 charge/discharge cycles at a current rate of 0.8 A g^−1^. SEM images in (**b**), (**d**) and (**f**) are magnified from the region outlined by dashed pink line in (**a**), (**b**) and (**c**) SEM images.

Secondly, different from the intercalation electrode materials (*i.e*. graphite or TiO_2_)^1^, the special conversion reaction of transition metal oxide and concomitant volume expansion/contraction would pulverize unstable structure and lead to poor cycling stability^18^,^19^,^20^,^21^,^22^,^23^,^24^. Supplementary Fig. S5 shows the representative cyclic voltammograms (CVs) of QNHM based LIBs. During the first cathodic sweep, a strong peak located around 0.7 V can be ascribed to the irreversible decomposition of Ni_x_Co_3-x_O_4_ to metallic Ni and Co, and then the reduction peak shifts to a higher potential at 1.12 V in the subsequent cycles. While the lithium extraction process is characterized by two anodic peaks at around 1.67 V and 2.07 V, which is associated with the oxidation of Ni^0^ to Ni^2+^ and Co^0^ to Co^3+^, respectively^15^,^21^. The above conversion reaction can be furthered confirmed by XRD measurements that carried out at fully discharge and charge state in the first cycle (Supplementary Fig. S6). With discharge voltage approaching 0.01 V, there was no obvious diffraction peaks, illustrating the formation of amorphous metallic Ni, Co, and Li_2_O matrixes. While the battery was charge back to 3 V, the Co_3_O_4_ or Ni_x_Co_3-x_O_4_ peaks emerged obviously, suggesting the conversion reaction is reversible. Based on the above analysis, we can imagine that how difficult Ni_x_Co_3-x_O_4_ materials can maintain their initial architecture due to the above conversion reaction. To further confirm the structure stabilizing effect of QNHM on long life electrochemical performance, we performed the SEM and XRD characterization of QNHM, square microtube, and circular microtube electrodes after completions of 100 charge/discharge cycles at a current rate of 0.8 A g^−1^. As we expected, QNHMs can still maintain their initial structure after completing severe cycles (Fig. 7a,b), but the square microtubes (Fig. 7c,d) and circular microtubes (Fig. 7e,f) seriously collapsed into the aggregation of irregular particles. As shown in Supplementary Fig. S7, the XRD measurements demonstrate that the peaks of Co_3_O_4_ phase were still detected in QNHMs after 100 cycles, indicating the well preserved crystal structures. However, the irregular particle aggregation derived from the square and circular microtubes were changed into amorphous matrixes since no obvious peaks of Co_3_O_4_ crystal were detected. As discussed in previous Fig. 1 and Fig. 3, the circular and square microtubes consist of loosely aggregated irregular spherical and polyhedral particles, yet our QNHMs are composed of continuously twinned submicron cubes, which are very stable and can be well preserved during charge-discharge cycle.

## Conclusions

In summary, we have presented for the first time the successful synthesis of quasi-single-crystal Ni_x_Co_3-x_O_4_ structure constructed by continuously twinned submicron cubes. Benefiting from such unique structural features, we also have demonstrated a high-performance and stable LIB by using QNHMs as electrode design that can both provide efficient electron pathway and suppress the electrode pulverization. At current density of 0.8 A g^−1^, it delivered reversible discharge capacities as high as 1470 mAh g^−1^ over 100 cycles and 590 mAh g^−1^ even after 1000 cycles. In addition, SEM and XRD characterization further reveal that the QNHMs can stabilize the structure after enduring the severe convention reaction cycles. Intriguingly, we have also synthesized a series of Ni_x_Co_3-x_O_4_ products with systematic shape evolution including circular microtubes consisted of spheres, square microtubes constructed by polyhedra, as well as monodisperse particles such as spheres, polyhedra, and cubes. On the basis of above structure and performance analyses, the advantages of our QNHMs applied in lithium ion batteries are fourfold: (1) submicron-cubes are continuously twinned into a very stable quasi-single crystal structure for enduring large volume expansion and suppressing pulverization of the anode during cycles, which is beneficial to the cycling stability. (2) The quasi-single-crystal structure can provide a continuous charge transport pathway, which is indispensable for good electrochemical performance. (3) One dimensional micron-sized tubular structure would decrease electrolyte diffusion resistance and serve as a buffering reservoir of electrolyte. (4) The submicron cubes served as the primary building blocks could offer a short solid-phase ion diffusion length to speed up the ion transport. These findings provide a comprehensive understanding of effects of crystal plane, specific surface area and stable electrode structure design on the electrochemical performance of LIBs. It is also important to note that such quasi-single-crystal structure may be helpful to explore other high-energy lithium storage materials based on alloying or conversion reactions.

## Method

### Materials Synthesis

All chemical reagents were commercially available and used without further purification. For the synthesis of Ni_x_Co_3-x_O_4_ products with systematic shape evolution from circular microtubes to square microtubes and QNHMs, 0.8 g Ni(NO_3_)_2_∙6H_2_O (A.R., Aladdin) and 3.2 g Co(NO_3_)_2_∙6H_2_O (A.R., Aladdin), were added to 60 mL of 8.3% (w/w) ammonia solution under vigorous stirring for 30 min. The reaction solution was then transferred to a 100 mL Teflon-lined autoclave and hydrothermally heated at 180 °C, 200 °C, and 230 °C for 24 h, respectively. Effects of reaction time on the morphologies of Ni_x_Co_3-x_O_4_ hexagonal microtubes were also investigated at hydrothermal temperature of 230 °C for 1–24 h. A series of monodisperse Ni_x_Co_3-x_O_4_ particles were also produced by justly decreasing concentration of Ni and Co precursor, increasing ammonia concentration, and adding ployvinylpyrrolidone (PVP) surfactant. To be specific, 0.4 g Ni(NO_3_)_2_∙6H_2_O, 1.6 g Co(NO_3_)_2_∙6H_2_O and 0.1 g PVP were added to 60 mL of 25% (w/w) ammonia solution. The monodisperse Ni_x_Co_3-x_O_4_ particle such as spheres, polyhedra, and cubes were obtained at hydrothermal temperature of 230 °C with different reaction time of 3 h, 12 h, and 24 h, respectively. The autoclave was taken out and cooled naturally to room temperature. After that, the precipitate was collected and washed with ethanol and deionized water for several times, and dried at 60 °C for 24 h. Finally, the obtained samples were calcined at 550 °C for 3 h with a heating rate of 1 °C min^−1^ before it was used as anode material in LIBs.

### Structural characterization

The morphology of the samples was examined using the thermal field-emission environmental scanning electron microscope (Quanta 400F, FEI/OXFORD/HKL, Dutch). The crystal structure analysis was conducted by XRD instruments (D/Max-IIIA, Rigaku, Japan) using Cu-Kα radiation (λ = 1.5418 Å). The TEM, high-resolution TEM images, and SAED were obtained with a JEOL JEM- 2010HR transmission electron microscope operating at 200 kV. Chemical composition of QNHMs was analysed by X-ray photoelectron spectroscopy (ESCALAB 250, Thermo-VG Scientific). The pore characteristics and specific surface area were measured by a nitrogen adsorption–desorption apparatus (ASAP 2020M, Micromeritics).

### Electrochemical measurements

The electrochemical measurements were performed using two-electrode CR2025-type coin cells with pure lithium metal serving as both the counter and reference electrodes at room temperature. The working electrode was composed of active material (*e.g*., QNHMs, square microtubes, circular microtubes, monodisperse spheres, polyhedral, and cubes), conductivity agent (carbon black, Super-P-Li), and binder (polyvinylidene difluoride, PVDF, Aldrich) in a weight ratio of 5:3:2 in N-methyl-2-pyrrolidone (NMP). The electrolyte used was 1.0 M LiPF_6_ in a 1:1 (w/w) mixture of ethylene carbonate-diethyl carbonate (Technologies, USA). A porous membrane (Celgard 3400) was used as a separator. Cell assembly was carried out in an Argon-filled glovebox (Mikrouna) with moisture and oxygen concentrations below 1.0 ppm. The cyclic voltammetry (CV) measurements were scanned at 0.005 mV S^−1^ in a voltage window of 0.01–3.0 V on a CHI760C electrochemical workstation. Galvanostatic charge/discharge cycling was carried on with a NEWARE battery testing system with a voltage window of 0.01–3.0 V at various current densities of 0.8–4.0 A g^−1^.

## Additional Information

**How to cite this article**: Zhou, Y. *et al*. Controlled synthesis of series Ni_x_Co_3-x_O_4_ products: Morphological evolution towards quasi-single-crystal structure for high-performance and stable lithium-ion batteries. *Sci. Rep*. **5**, 11584; doi: 10.1038/srep11584 (2015).

## Supplementary Material

Supplementary Information
