# Supplementary material for: Controlled synthesis of series NixCo3-xO4 products: Morphological evolution towards quasi-single-crystal structure for high-performance and stable lithium-ion batteries
Source: Sci Rep. 2015 Jun 24;5:11584. doi: 10.1038/srep11584 (PMC4478471; doi:10.1038/srep11584)
Supplement: Supplementary Information [file srep11584-s1.doc]

Supplementary Information

Controlled synthesis of series Ni_x_Co_3-x_O_4_ products: Morphological evolution towards quasi-single-crystal structure for high-performance and stable lithium-ion batteries

Yu Zhou^1^, Yong Liu^1, *^, Wenxia Zhao^2^, Hai Wang^3^, Baojun Li^1^, Xiang Zhou^1^ & Hui Shen^1^

^1^School of Physics and Engineering, State Key Laboratory of Optoelectronic Materials and Technologies, Sun Yat-sen University, Guangzhou 510275, China

^2^Instrumental Analysis & Research Center, Sun Yat-sen University, Guangzhou 510275, China

^3^ Key Laboratory of New Processing Technology for Nonferrous Metal and Materials, Ministry of Education Guilin University of Technology, Guilin 541004, China

*Corresponding author*:* liuyong7@mail.sysu.edu.cn


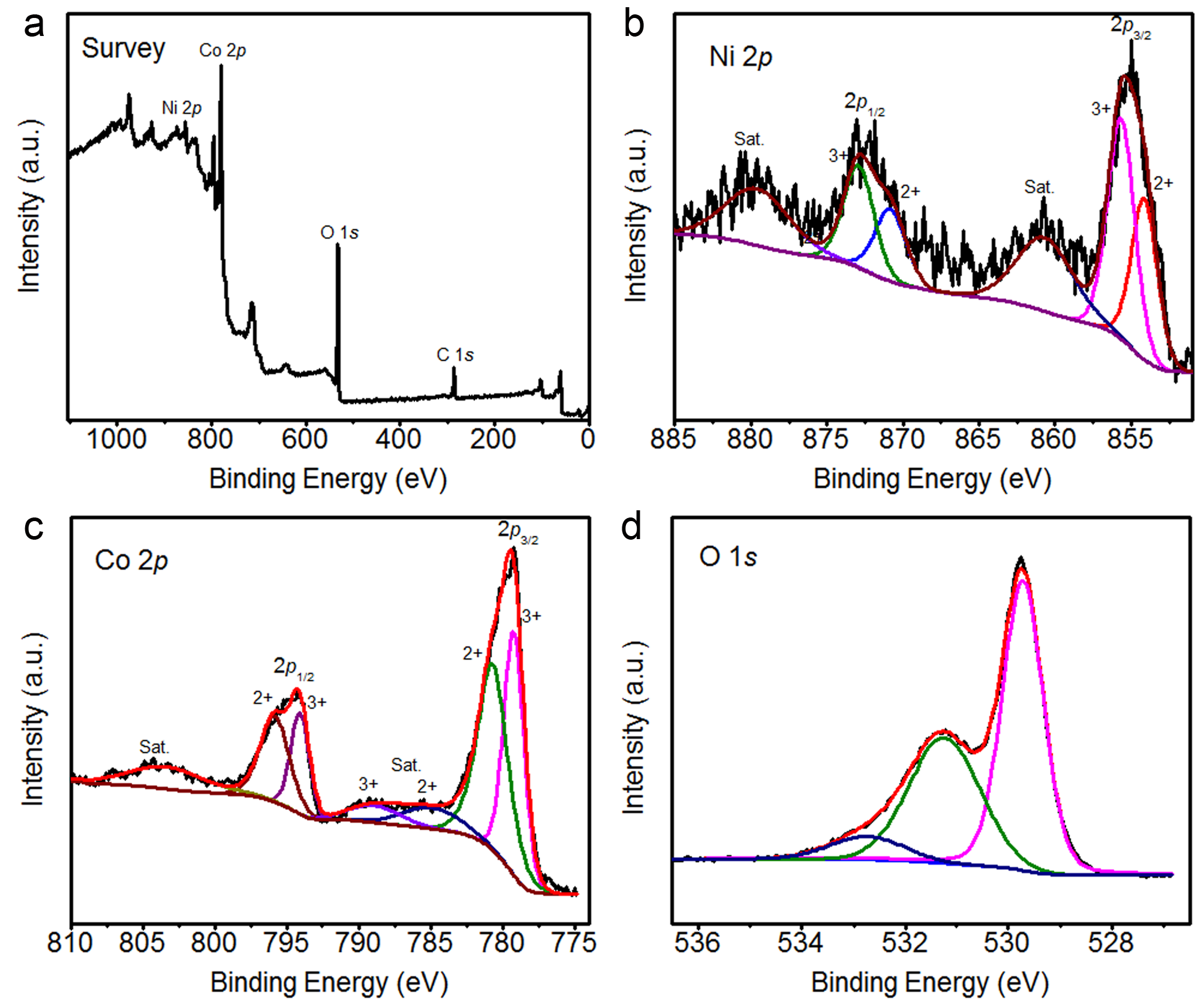


**Figure S1****.** XPS spectrum of QNHMs. (a) Survey spectrum, (b) Ni 2p core level, (c) Co 2p core level, and (d) O 1s core level.


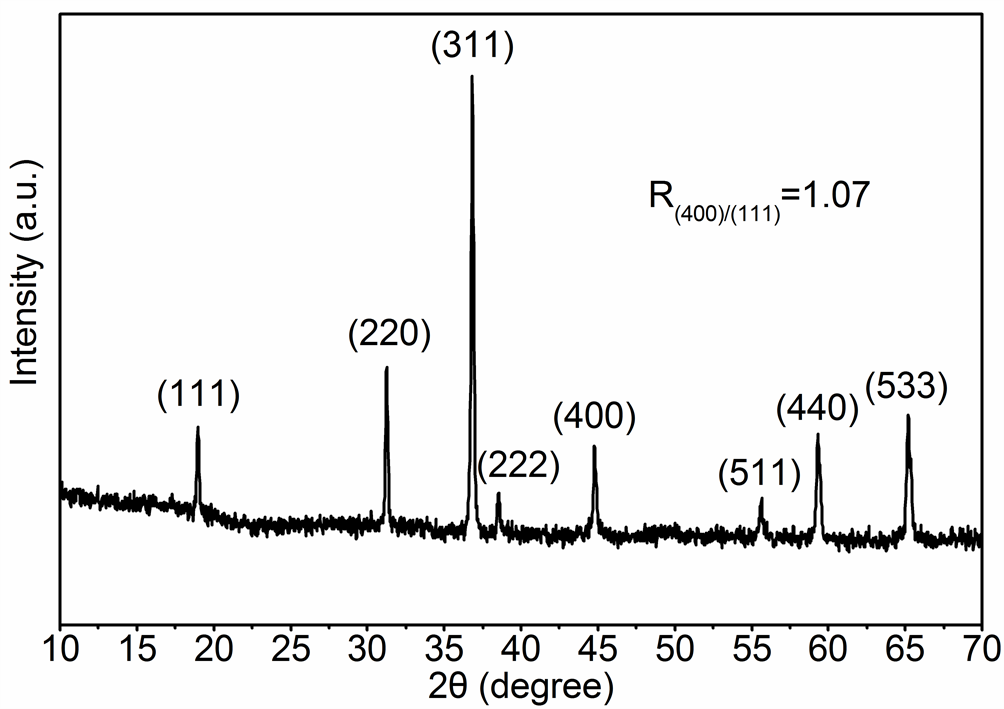


**Figure S2.** XRD pattern of QNHMs calcined at 550 °C for 3 h.

**
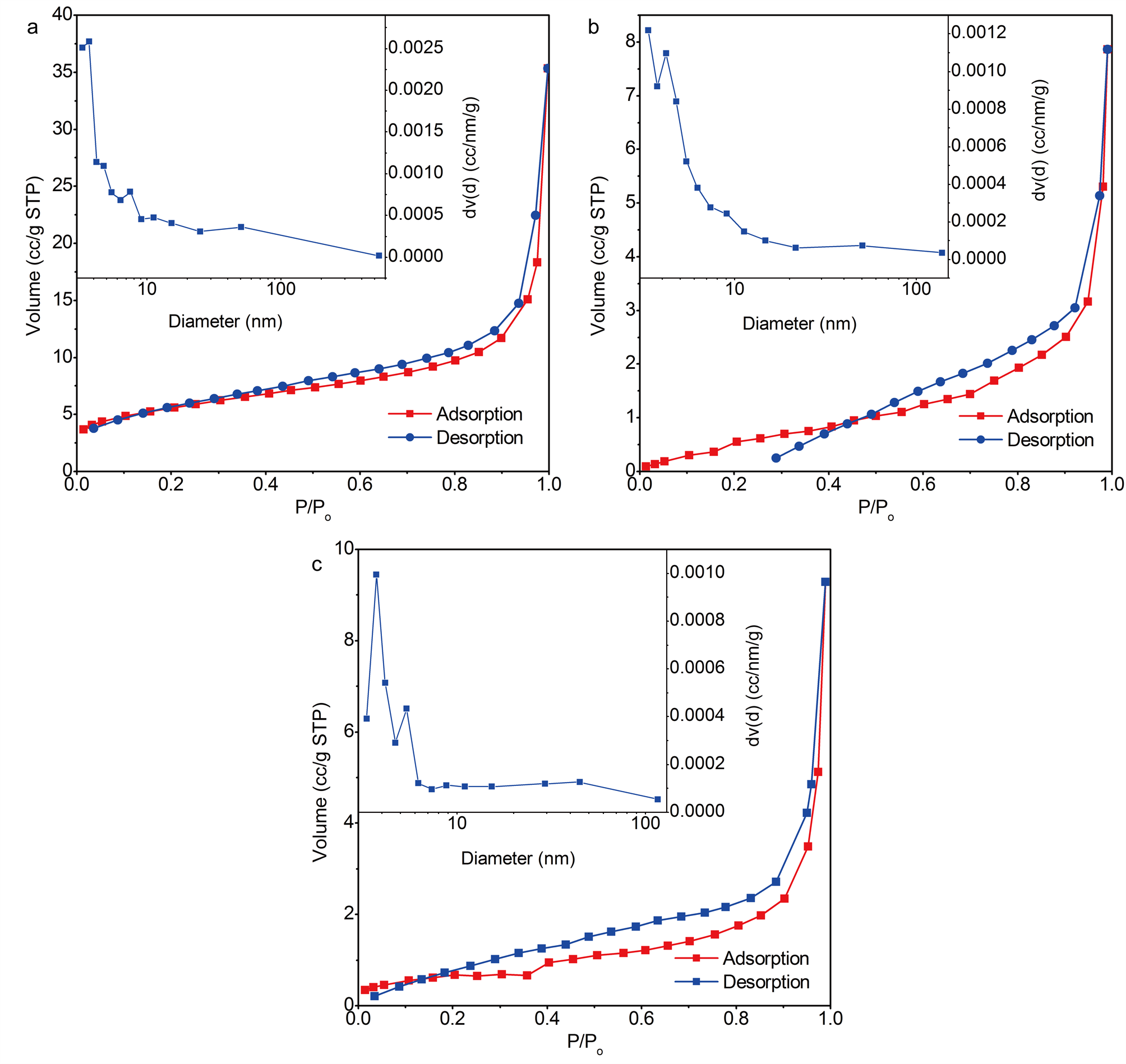
**

**Figure S3.** N_2_ adsorption-desorption isotherms for (a) circular microtubes, (b) square microtubes, and (c) QNHMs, respectively. The inset shows their corresponding pore size distribution.

**
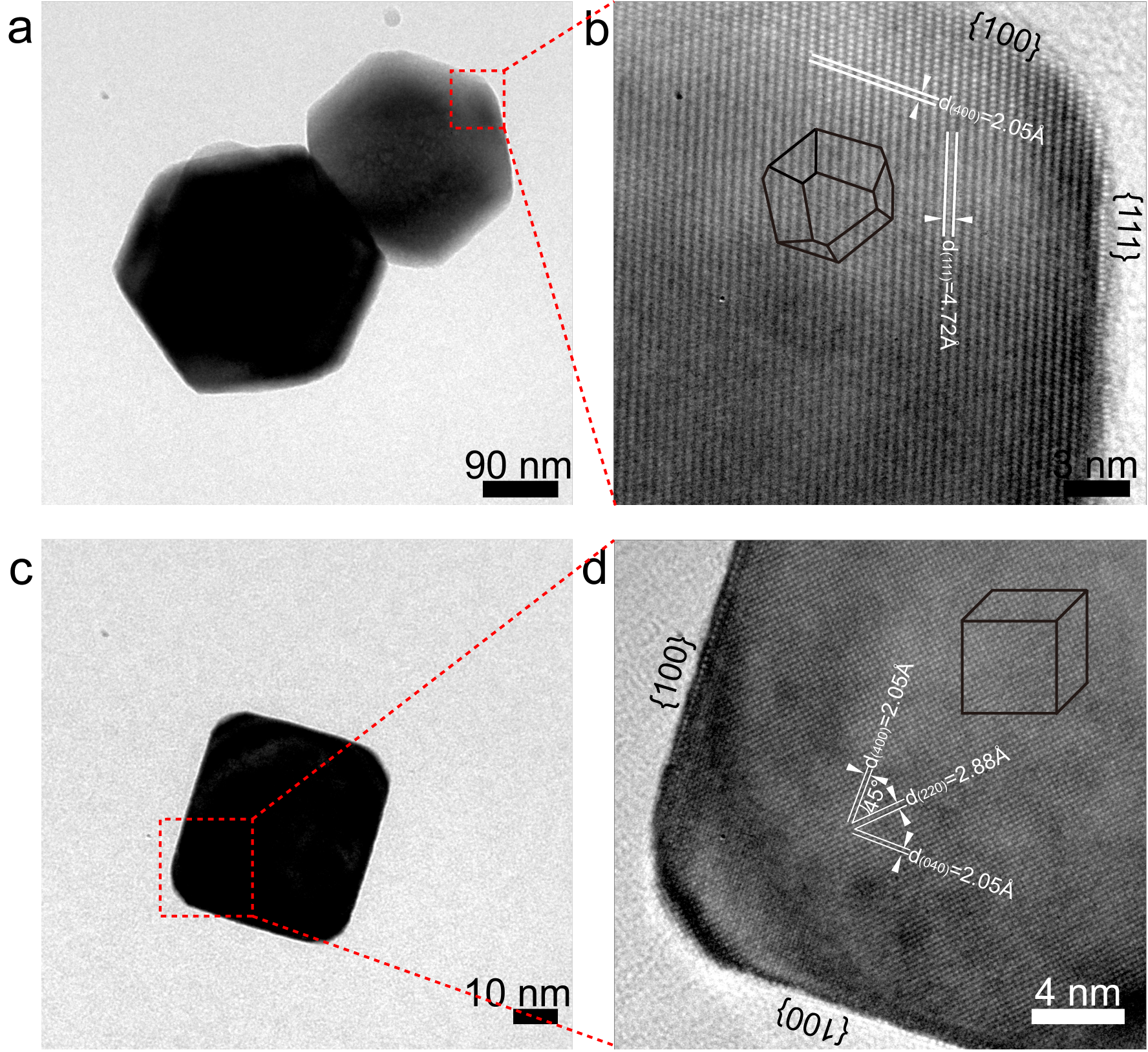
**

**Figure S4.** (a) TEM image and (b) HRTEM image of the Ni_x_Co_3-x_O_4_ polyhedra. (c) TEM image and (d) HRTEM images of the Ni_x_Co_3-x_O_4_ cubes.


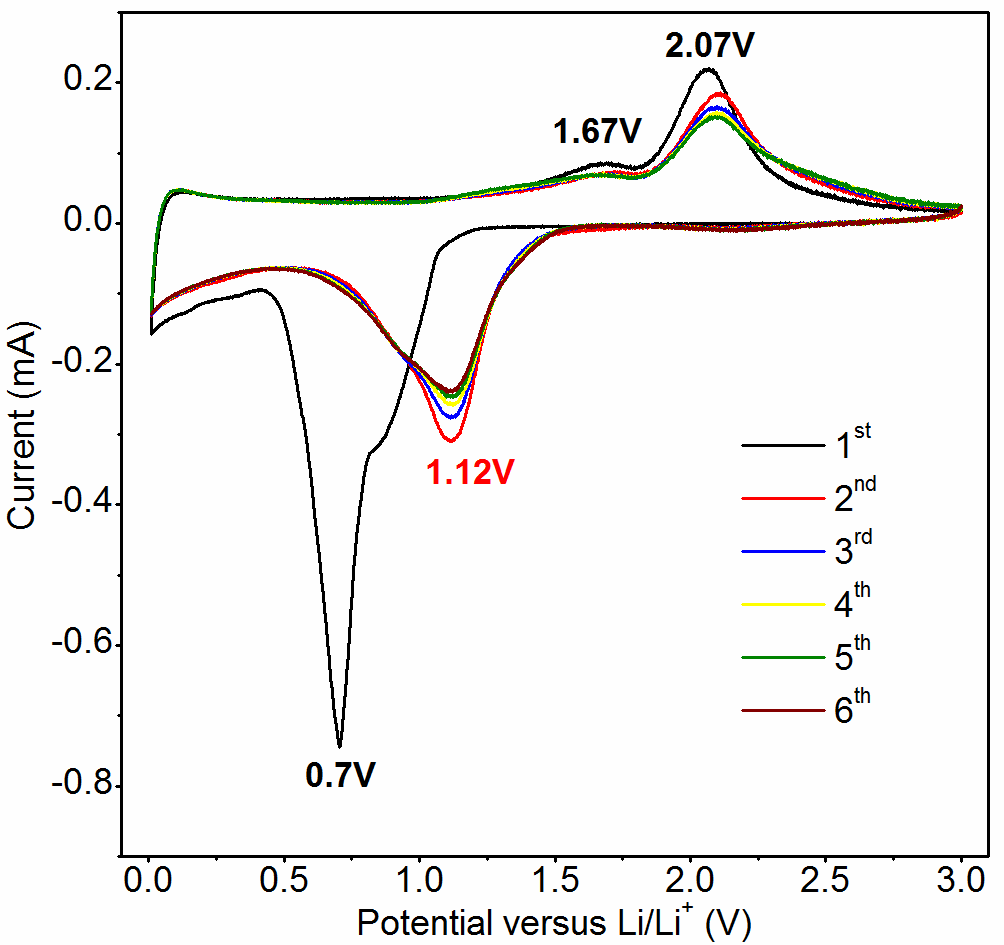


**Figure S****5.** The cyclic voltammograms of QNHM based LIBs obtained from the first cycle to the sixth cycle at a scan rate of 0.005 mV S^-1^ in the voltage range of 0.01-3 V *versus* Li/Li^+^.


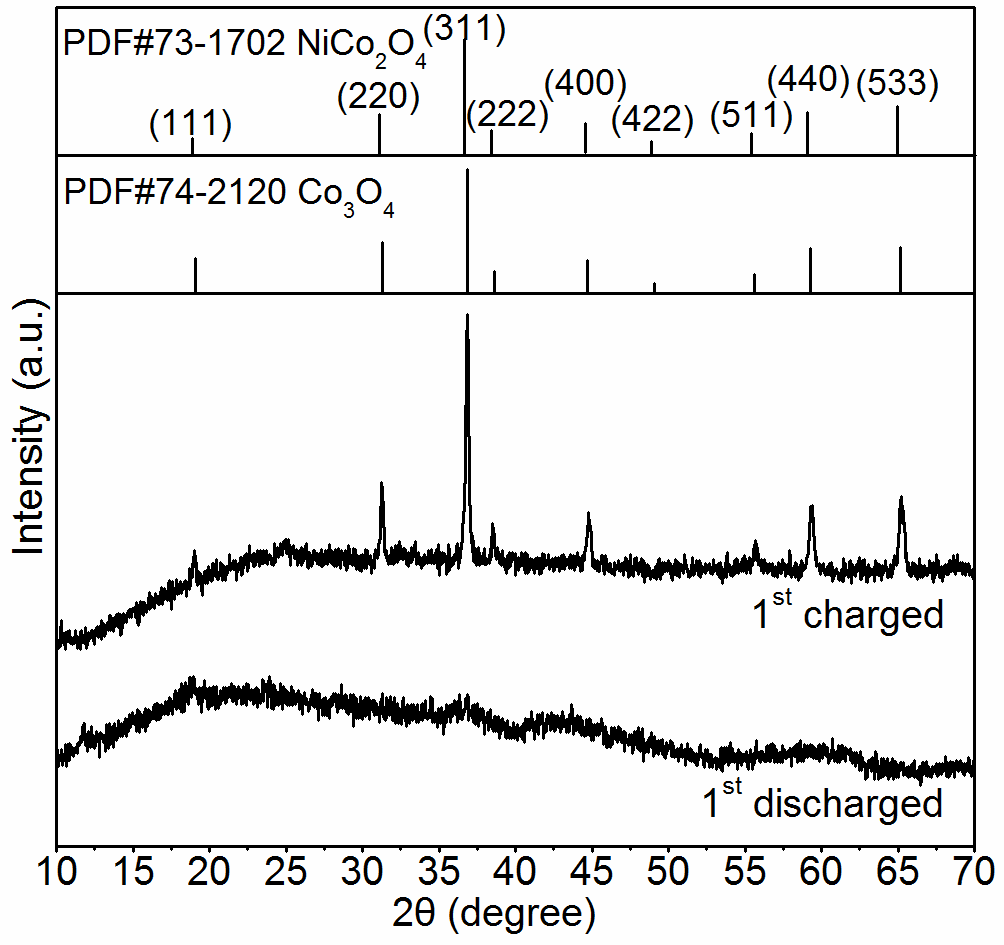


**Figure S6.** XRD patterns of QNHMs based electrodes measured at the fully discharged and charged state in the 1^st^ cycle at a current rate of 0.8 A g^-1^.

**
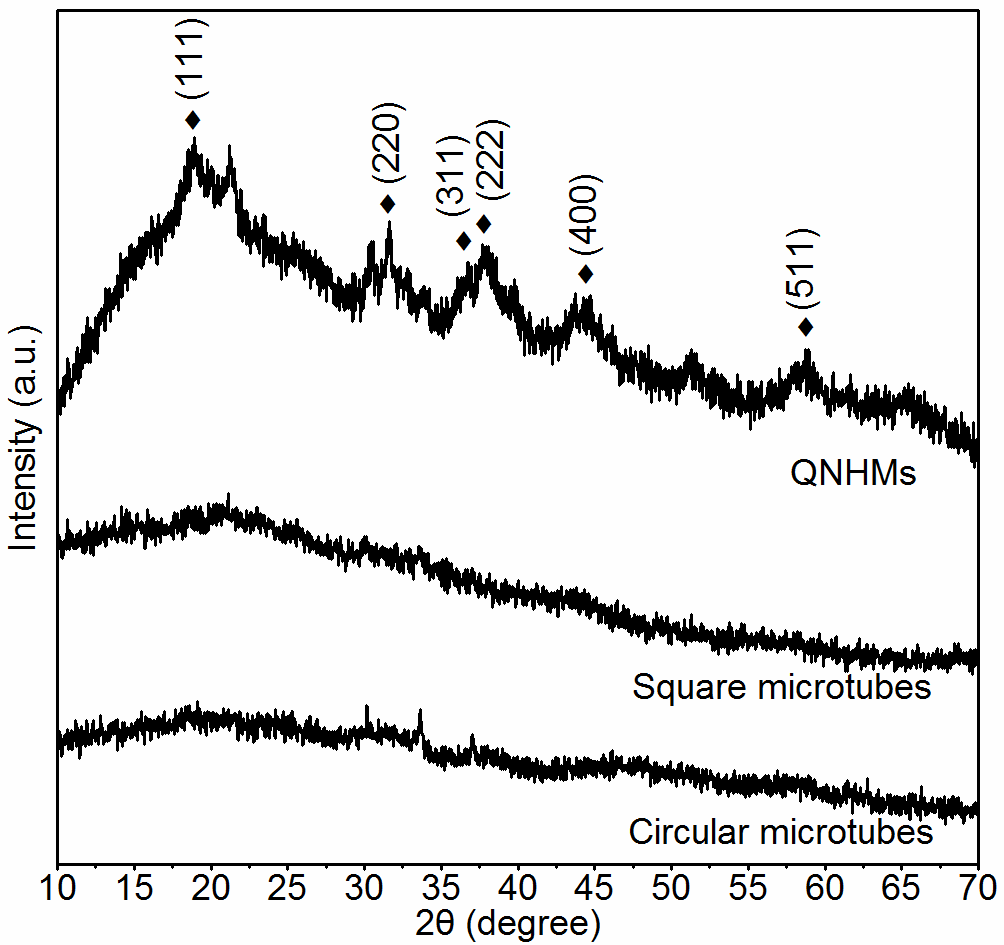
**

**Figure S7.** XRD patterns of QNHM, square microtube, and circular microtube electrodes obtained after completions of 100 charge/discharge cycles at a current rate of 0.8 A g^-1^.
